# Gibraltar Epidemic of 1828

**Published:** 1831-01

**Authors:** H. Fraser

**Affiliations:** Chatham


### GIBRALTAR EPIDEMIC OF 1828.

*Letter from* Mr. Hugh Fraser, *late Surgeon to the Civil Hospital of Gibraltar. To the Editor.*

Sir: On my arrival here lately from Scotland, where I had been for some time, I found, in the Number of your Journal for November last, a letter addressed to Dr. James Johnson, by Dr. David Barry. That letter purports to be a reply to a paper written by me, in answer to what appeared in the Medico-Chirurgical Review of April last, as the summary of a paper read by Dr. Barry, before the College of Physicians, the preceding month.

My paper was written at Gibraltar in May 1830, and, when forwarded to Dr. Johnson, it was certainly my wish that it should be published *entire* in his Journal: however, after what has appeared on the subject from that gentleman, there can be no doubt as to his intentions in giving it to the public in the shape he did.

Although in possession of the most ample means of placing Dr. Barry, and many of his subtilities, in the full light of day, before the profession at large, this must be reserved for a future occasion; as, in the first place, I find that I am already too late for the insertion, in your next Journal, of the full details necessary for this purpose; and, secondly, knowing my man, I think it important, in order that the public may not be eternally told he has been misquoted or misunderstood, that Dr. Barry should furnish a copy of his paper, as read before the College of Physicians. If truth be his object in this controversy, he cannot refuse my demand that it be forthwith published, or lodged with you, so that I may have access to it. The Doctor cannot, with propriety, object to the publication or production of a paper, read in presence of

Reel: 81-26-2

so large an assembly, and the substance of which many might, if they had thought it worth while, have written down in short-hand. If he refuse, the public will know what conclusions to draw; but if he comply, I shall, as speedily as possible, furnish such materials as will show the public which side is likely to cry "*hold, enough!*"

On the points at issue in the history of this epidemic, it is impossible not to admit that honourable men, unacquainted with the *hole and corner work* which has taken place, must naturally feel surprised that persons, viewing the same facts, should give such discordant accounts of them; and to others, as well as to Dr. Johnson, some words and passages in my paper may have appeared acrimonious. To those, however, acquainted with the dark and odious proceedings that took place,—how facts, of the highest interest to science and to humanity, have, for certain purposes, been perverted,*—my language will only appear as just and proper indignation; and I am bold enough to consider that the public will be of the same opinion when all is known which should be known, as well from others as from me.

It was certainly my intention, when I wrote my paper, to show that, on a question of the most vital importance to nations, Dr. Barry was, in the first place, one of the last persons who should have obtruded statements on the public regarding the fever in question, for the simple reason, that he had not duly availed himself of even the few opportunities which he had had of forming opinions while present, which was only at the very close of the epidemic season: this I pledge myself to show, notwithstanding his manœuvres and mighty display of promised facts. In the next place, it was, as it is still, my intention to impugn his testimony on certain points, by showing how he had disfigured facts.

You may have perceived, Sir, it is true that, in these matters, I have marched directly and openly to the point,—while, in the paper of my adversary in reply; of him, who assured the public that nothing should induce him to descend to the vulgar weapon of acrimony, we have a document on record, than which there is no other, I believe, so disgraceful in the annals of medical controversy. It is difficult to say, whether petulance or acrimony prevail most. Then for foul inuendoes!

* I have no hesitation in saying, that Dr. Gillkrest, now Deputy Inspector of Hospitals, openly and loudly asserted, and proved before the commissioners appointed to inquire into the origin of the epidemic, that facts were attempted to be so perverted; and I can prove that the medical registers of the civil hospital were taken from me under the mask of—— (my fingers shall restrain what my pen was going to drop,) and afterwards withheld from me, for no other object than the perversion of truth.

inuendoes against whom? against those, Sir, whom, most assuredly, Dr. Barry shrinks from attacking openly. Better were it, indeed, for him, as I suspect he himself may now think, that his fingers had been burnt ere he had drawn up a composition so hideous as that must be held to be in the eyes of his department, and of the profession at large. If his jokes have not been already spoiled, I think he may look to their being so before this question is decided. He will discover, probably, if he has not done so already, that it is one thing to get up, for the amusement of his Gibraltar *camarilla*, an inflated production, full of arrogance and disgusting absurdities, and another to establish his patron's cause in a manner which may satisfy the public.

Dr. Barry says, that he expects a "*badgering*" from others. Never, let him be assured, have the evil doers among his countrymen of St. Giles's cried out, with more just cause, against the new police, than he has reason to cry out against those medical gentlemen of Gibraltar who maintain the cause of truth, having spurned the advantages which so obviously arose from attaching themselves, as Dr. Barry has done, to the cause of his friend the superintendent general of quarantine; which friend and zealous patron of Dr. Barry went out to Gibraltar, be it always remembered, at the close of the epidemic, and where his long-cherished creed of importation and contagion was (as also by Dr. Barry, *latterly*,) supported by him with a zeal not to be questioned.

I again demand that Dr. Barry should produce his paper, as read to the College of Physicians; and I remain, Sir, your obedient humble servant,

(Signed) H. FRASER.

*Chatham; Dec.* 18*th*, 1830.

---

